# Organizational interventions to implement improvements in patient care: a structured review of reviews

**DOI:** 10.1186/1748-5908-1-2

**Published:** 2006-02-22

**Authors:** Michel Wensing, Hub Wollersheim, Richard Grol

**Affiliations:** 1 Centre for Quality of Care Research (WOK), Radboud University Nijmegen Medical Centre, Nijmegen, The Netherlands

## Abstract

**Background:**

Changing the organization of patient care should contribute to improved patient outcomes as functioning of clinical teams and organizational structures are important enablers for improvement.

**Objective:**

To provide an overview of the research evidence on effects of organizational strategies to implement improvements in patient care.

**Design:**

Structured review of published reviews of rigorous evaluations.

**Data sources:**

Published reviews of studies on organizational interventions.

**Review methods:**

Searches were conducted in two data-bases (Pubmed, Cochrane Library) and in selected journals. Reviews were included, if these were based on a systematic search, focused on rigorous evaluations of organizational changes, and were published between 1995 and 2003.

Two investigators independently extracted information from the reviews regarding their clinical focus, methodological quality and main quantitative findings.

**Results:**

A total of 36 reviews were included, but not all were high-quality reviews. The reviews were too heterogeneous for quantitative synthesis. None of the strategies produced consistent effects. Professional performance was generally improved by revision of professional roles and computer systems for knowledge management. Patient outcomes was generally improved by multidisciplinary teams, integrated care services, and computer systems. Cost savings were reported from integrated care services. The benefits of quality management remained uncertain.

**Conclusion:**

There is a growing evidence base of rigorous evaluations of organizational strategies, but the evidence underlying some strategies is limited and for no strategy can the effects be predicted with high certainty.

## Introduction

Numerous studies have shown that at least 40% of the patients do not receive high-quality medical care [1]. So far, strategies to implement best evidence to improve clinical practice have been mainly targeted at improving the knowledge, attitudes and behaviors of healthcare workers [2]. Examples of these strategies are audit and feedback, reminder systems, educational meetings and educational outreach visits. These strategies appear to achieve a median of 10% absolute change of professional performance and no strategy is uniquely and consistently effective [3]. While this change may be clinically and economically relevant, further improvements are needed. Many patient outcomes are not only influenced by the performance of individual care providers, but also by the functioning of clinical teams and by broader organizational and financial structures. Contextual factors, such as a high burden of work or poor co-ordination mechanisms, can be important barriers for wide-scale and sustainable improvement [4]. Organizational changes could therefore offer important mechanisms for quality improvement.

Claims on the effectiveness of organizational strategies for improving the quality of care should be based on evidence from rigorous evaluations. While a number of reviews of specific organizational strategies have been published, no broad overview of research evidence on organizational strategies has been published. This paper focuses on organizational strategies, which could improve professional practice and health outcomes (Table 1). Decision makers need an overview of the evidence for their organizational measures in order to chose effective interventions and avoid ineffective interventions, yet the research literature on these strategies is scattered over a large number of journals. This paper aims to provide a structured review of the research evidence from systematic literature reviews of organizational interventions with respect to their effects on professional performance, patient outcomes and costs.

**Table 1:** Organizational changes to improve patient care

- Revision of professional roles: Change of tasks and responsibilities of health professionals, such as increased medical roles to nurses and enlarging the roles of pharmacists.
- Multidisciplinary teams: Clinical teams or collaborations of physicians, nurses and allied health professionals to improve professional performance and patient outcomes.
- Integrated care services: Organized systems for care delivery (also labeled as disease management programs or integrated care pathways) to patients with specific diseases, who receive care according to a protocol, which covers the spectrum from screening to education, treatment and monitoring. Case management overlaps with disease management and has been included in the category.
- Knowledge management: Knowledge management is the optimal organization of knowledge within an organization. In practice, it mainly refers to the use of information and communication technology to support patient care, such as computerized medical record keeping.
- Quality management: A management approach, which focuses on customers, continuous efforts to improve, measurement and analysis of performance, and supportive leadership and organizational culture. Various approaches, such as total quality management, continuous quality improvement, and business redesign are included in this category.

## Methods

### Data sources

We performed searches in Pubmed (1994–2003) and the Cochrane Library (accessed in January 2004). Older reviews were excluded, because their validity for current decision makers may be limited and we assumed that the reviewed studies would be included in later reviews. The search strategy in Pubmed combined the MeSH terms 'review literature' and 'meta-analysis' with the MeSH term 'healthcare quality'. The search in the Cochrane Library focused on reviews of the Cochrane Effective Practice and Organization of Care (EPOC) Group. We checked references in identified papers in previous overviews of systematic reviews in this field, which were themselves based on exhaustive systematic searches [5-7]. Authors' personal literature collections were also examined; these were partly based on manual searching in health services research journals over the last 10 years. Only papers written in English were included. Our search was not designed to be comprehensive, but to provide a comprehensive overview of the available research evidence.

### Study selection

A review was included if it reported on its search strategy, if it focused (at least partly) on rigorous evaluations of organizational strategies (defined as planned re-arrangements of one or more aspects of the organization of patient care), and if it was published in 1995 or later. Rigorous evaluations comprised randomized trials, interrupted time-series, controlled before-and-after studies, and prospective comparative observational studies. Papers were included by the first author and the inclusion was checked by the second author. Some reviews also included studies on non-organizational strategies or non-rigorous studies; these sections in the reviews were not used. We did not include reallocation of services from hospital to primary care settings.

### Data extraction

A taxonomy of organizational strategies to improve patient care was developed to organize the results (Table 1); this was consistent with other lists of organizational interventions such as that used by EPOC. Two researchers extracted from the reviews information on their focus, methodological quality, and main results. The number of studies reported in the table refers to the number of rigorous evaluations of organizational interventions; this may be lower than the total number of studies in the review as we focused only on rigorous studies of organizational interventions. Two authors independently assessed the methodological quality of the review, as opposed to the included studies, using a previously used 9-item scale (we did not use the summary assessment in the original scale) [7]. A score of 7 or more was taken to indicate good methodological quality. The summaries of the main results regarding effects on professional practice, patient outcomes and costs were based on the text in the original papers, derived from the abstract, results section or discussion section, focusing on quantitative summaries when available.

### Data synthesis

We expressed effects in terms of average effect size (AES), standardized mean difference (SMD), weighted mean difference (WMD), adjusted odds ratio's (AOR), adjusted relative risk (ARR), median net change (MNC) or percentage studies with improvements (PSI). Except for PSI all figures were extracted from the papers. If a meta-analysis had been performed, we also recorded whether the effect was significant (S) or not (NS). If quantitative summary measures of effectiveness were not used, the range of effects across studies was used. If this was not available, the authors' main qualitative conclusions were reported.

## Results

### Description of studies

A total of 36 reviews were included [8-44], of which 21 were of good methodological quality. The reviews with lower scores for methodological quality had not used optimal procedures for data-extraction and data-analysis. The studies were too heterogeneious regarding strategies and context factors to allow statistical pooling; furthermore, information on contextual factors was very limited.

### Revision of professional roles (Table 2)

**Table 2:** Revision of professional roles

Author, number of studies	Quality score	Focus	Main results
Beney 2000 N = 16	7	Enlargement of the role of the public pharmacist	Changed use of healthcare services (PSI 6/6 = 100%). Improved patient outcomes (PSI 10/13 = 77%). No change in: quality of life.
Bower 2000 N = 38	9	Mental health workers in primary care: replacement of/consultation to primary care providers	*Replacement: lowered consultation rates (PSI 2/8 = 25%), short term reduction on psychotropic prescribing (PSI 4/11= 36%), long term changes psychotropic prescribing (PSI 3/6 = 50%), reduced mental health referrals (PSI 3/6 = 50%). *Consultation: more appropriate short term prescribing (PSI 3/6 = 50%). No change in: consultation rates, referral patterns.
Brown 1995 N = 13	4	Nurse practitioners in primary care	Improved laboratory testing (AES 0.20), resolution of pathological conditions (AES 0.28), patient satisfaction (AES 0.30). No change in: quality of care, prescribing, functional status, consultation rates, use of emergency service.
Dijkstra 2004 N = 13	7	Revision of professional roles for guideline implementation in hospitals	Improved professional performance (AOR 9.78, S).
Horrocks 2002 N = 11	6	Nurse practitioners in primary care	Improved patient satisfaction (SMD 0.27), longer consultations (WMD 3.67 minutes), more investigations (OR 1.22). No change in: health status.
Loveman 2003 N = 6	8	Specialist nurses in diabetes mellitus	No change in: HbA1c, emergency admissions, quality of life.
Stone 2002 N = 20	6	Organizational change (mainly involvement of non-physician staff and clinics devoted to prevention) to improve adult immunization and cancer screening	Improved preventive activities (AOR range 2.74 – 17.6).
Smith 2001 N = 4	7	Outreach nursing for chronic obstructive pulmonary disease	Increased hospital service utilization (PSI 2/2 = 100%). No change in: mortality, lung function, health related quality of life.
Thompson 2003 N = 7	8	Dietary advice by dietitians compared to self-management materials.	No change in: patient outcomes.

Nine reviews focused on revision of professional roles, of which five were of good methodological quality. All focused on revised roles for non-physicians.

An older review identified 13 (quasi-) randomized trials, which compared nurse practitioners to physicians in primary care [13]. It found that quality of care, resolution of pathological conditions and functional status were not affected, while number of tests ordered and patient satisfaction increased. Similar findings were reported in a more recent review [19]. This latter review also reported that nurse practitioners had longer consultations, while prescriptions, return consultations and referrals did not differ. A review that focused on the effect of specialist nurses in diabetes care found that glycated hemoglobin was not different from usual care over a 12-month period [2]. Outreach nursing in patients with chronic obstructive pulmonary disease did not change patient outcomes, but it increased the use of hospital services [35].

A broad review on quality improvement in hospitals identified 13 studies on improvement strategies, which comprised the component of revision of professional roles [15]. This component significantly contributed to improved professional performance in a meta-regression analysis. A review on adult immunization and cancer screening found 20 trials, which included a component of organizational change – mainly designation of specific prevention responsibilities to nonphysician staff [37]. The meta-regression analysis showed that changing roles was one of the most effective intervention components in increasing use of the clinical and preventive services (compared to educational approaches, feedback and reminding strategies).

A review on enlarged roles of outpatient pharmacists (15 randomized trials, one controlled trial) showed that delivery of pharmacist services influenced the use of services, prescribing patterns, and patient outcomes [11]. Effects on costs were uncertain. Mental health workers replacing primary care providers did not consistently change psychotropic prescribing, consultation rates or mental health referrals [12]. There was some evidence that consultation with primary care providers by mental health workers had a direct effect on prescribing behavior when used as part of complex, multifaceted interventions [12]. A review of advice given by dietitians showed that dietitians did not affect blood cholesterol more than self-help resources [39].

Overall, it seems that revision of professional roles can improve professional performance, while positive effects on patient outcomes remain uncertain. Revision of roles seemed especially effective in preventive care, but the effect in relation to specialized nurses in primary care are still unresolved.

### Multidisciplinary teams (Table 3)

**Table 3:** Multidisciplinary teams

Author, number of studies	Quality score	Focus	Main results
Hearn 1998 N = 5	5	Palliative care teams in advanced cancer	Improved patient and carer satisfaction (PSI 4/5 = 80%). Improved pain and symptom control (PSI 80%). Reduced hospital stay and overall costs (PSI 4/5 = 80%).
Mitchell 2002 N = 7	6	Arrangements that linked family physicians to specialist practitioners	Improved clinical behavior (PSI 4/4 = 100%). Cost savings (PSI 1/2 = 50%). No change in: health outcomes.
Philbin 1999 N = 2	4	Multidisciplinary teams for patients with congestive heart failure	Improved quality of life (PSI 1/2 = 50%). Reduced use of medical care (PSI 1/2 = 50%).
Vliet Vlieland 1997 N = 15	4	Multidisciplinary teams caring for rheumatoid arthritis	Inpatient teams versus usual outpatient care: improved short-term disease activity (PSI 4/4 = 100%), increased costs (2/2 = 100%). Outpatient teams versus usual outpatient care: improved disease outcomes (PSI 2/5 = 40%).
Zwarenstein 2000 N = 2	7	Interventions to promote collaboration between nurses and doctors	Reduced hospital stay (PSI 1/2 = 50%). No change in: mortality.

Five papers looked at studies on various interventions to enhance multidisciplinary collaboration, of which one was of good methodological quality [44].

In a review of palliative care teams four of the five randomized trials found that the co-coordinated specialist approach resulted in similar or improved outcomes in terms of patient and family satisfaction, anxiety, pain and symptom control [18]. Those studies that examined costs showed a tendency to reduce hospital days and equal or lower costs.

The involvement of a primary care practitioner in a specialist team was examined in a review, which identified seven randomized trials on programs for chronic or complex conditions [28]. While there were mixed effects for patient outcomes, they improved clinical performance of primary care providers, higher patient knowledge and higher patient satisfaction. Two studies examined costs, showing mixed effects. Only two randomized trials were identified in a review on interventions to promote collaboration between nurses and doctors [44]. These showed reduced hospital stay without change of mortality.

A review on multidisciplinary teams for congestive heart failure patients identified two randomized trials, which showed similar or improved outcomes [31]. Results regarding use of hospital care were inconsistent. The review on multidisciplinary teams for rheumatoid arthritis patients comprised 15 controlled trials (nine of which were randomized) [40]. The six trials of inpatient teams compared with regular outpatient care showed greater improvements in disease activity and in functional status immediately after treatment, which diminished over time. Five of the six trials on outpatient teams showed improvements on various patient outcomes compared with regular outpatient care.

Overall, it seems that multidisciplinary teams can improve patient outcomes. They have primarily been tested in highly prevalent chronic diseases.

### Integrated care services (Table 4)

**Table 4:** Integrated care services

Author, number of studies	Quality score	Focus	Main results
Badamgarav 2003 N = 11	7	Rheumatoid arthritis	Improved functional status (AES 0.27 NS).
Ferguson 1998 N = 9	4	Case management in various patient populations	Improved patient-centered outcomes (PSI 6/6 = 100%), improved clinical outcomes (PSI 2/2 = 100%), reduced health resource use PSI 2/7 = 29%).
Kwan 2001 N = 10	9	In-hospital pathways for stroke	Fewer urinary tract infections (AOR 0.38, S). Fewer readmissions (AOR 0.11, S). More computer tomography brain scans (AOR 3.66, S). More carotid duplex studies (AOR 2.45, S). Reduced patient satisfaction (P = 0.02). Reduced quality of life (P = 0.005). No change in: mortality, dependency, or discharge destination.
McAllister 2001 N = 11	7	Disease management for heart failure in patients discharged from hospital	Decreased hospital use (ARR 0.87), cost savings (PSI 7/8 = 88%). No change in: all-cause mortality.
McAllister 2001 (BMJ) N = 12	7	Secondary prevention of coronary heart disease in outpatients	Reduced hospital use (ARR 0.84 S), improved quality of life/functional status (PSI 5/8 = 63%), cost savings (PSI 2/3 = 67%). No change in: all-cause mortality, recurrent myocardial infarction.
Norris 2002 N = 42	5	Disease management and case management in diabetes	Disease management: improved professional performance (SMD range 10–30%). Improved glycated hemoglobin (MNC -0.5% S). Case management: improved glycated hemoglobin (MNC -0.53% S).
Ram 2001 N = 1	9	Asthma clinics in primary care	Improved peak flow scores and other patient outcomes (PSI 1/1 = 100%).
Stroke Unit Trialist Collaboration 1997 N = 19	6	Organized inpatient care after stroke (rehabilitation, staff specialization, training and staff education)	Reduced mortality (AOR 0.83, S). Reduced dependency or mortality (AOR 0.69, S). Reduced institutionalization (AOR 0.75, S). Reduced length of hospital stay (ARR 0.92 S).
Weingartenn 2002 N = 102	6	Disease management programs for patients with chronic illness: A. Provider education, feedback and reminders. B: Patient education, reminders and financial incentives.	A: provider adherence to guidelines (AES range: 0.44 – 0.61), patient disease control (AES range: 0.17 – 0.35). B: patient disease control (AES range: 0.24 – 0.40).

Eight reviews focused on integrated services, of which five were of good methodological quality.

A review on stroke considered organized in-patient care, including both dedicated stroke units and mixed assessment/rehabilitation units. It included 19 trials (12 randomized), and showed favorable effects of stroke care [38]. A second review on stroke focused on in-hospital pathways, which were described as 'protocols for well-organized multidisciplinary care' [21]. It identified three randomized trials and seven other studies, which showed no differences regarding objective outcomes, but deterioration of patient reported outcomes.

A review on ambulatory patients with heart failure (11 randomized trials) found that these reduced hospitalization but not all-cause mortality [25]. The programs were cost saving in most studies that reported cost data. A review on secondary prevention programs in coronary heart disease (12 randomized trials) found largely the same results, although only three studies examined costs [26]. There were several studies that showed improved quality of life and functional status in patients from disease management groups.

A review of diabetes care showed improved glycated hemoglobin levels in both disease management (17 studies in a meta-analysis) and case management (11 studies) [29]. The improvement was similar when case management was delivered in addition. Disease management in rheumatoid arthritis had a small non-significant overall effect on functional status [8]. Longer programs or programs with more components were not consistently more effective.

A review on case management programs in primary care (nine randomized trials) focused on comprehensive programs and various conditions, including asthma, congestive heart failure, diabetes, and geriatric conditions [16]. Positive effects were found on patient-centered and clinical outcomes, but not on use of resources. The review on asthma [32] identified only one randomized trial, which showed some improvements in health outcomes.

An extensive review of controlled trials regarding disease management in chronic illness examined the effects of interventions, used within disease management programs [43]. The programs included a wide variety of interventions. While the interventions themselves were not organizational, only applications in the context of organized care for chronic illness were considered. It showed that both provider-directed interventions and patient interventions were associated with effects on provider adherence to guidelines and disease control.

Overall, integrated care systems can improve patient outcomes and save costs. They have been extensively tested in highly prevalent chronic conditions.

### Knowledge management (Table 5)

**Table 5:** Knowledge management

Author, number of studies	Quality score	Focus	Main results
Balas 1996 N = 100	6	Computerized information services in different settings. A. provider prompt, B. provider feedback, C. computerized medical record, D. assisted treatment planning, E. computerized patient education.	Improved test ordering/prevention in A (PSI 14/16 = 88%), B (PSI 7/9 = 78%), and C (PSI 6/8 = 75%). Improved drug prescription in D (PSI 10/12 = 83%). Improved patient knowledge in E (PSI 8/9 = 89%).
Balas 2004 N = 40	7	Computerized knowledge management in diabetes care. A. provider prompt, B. home glucose records	Improved guideline compliance in A (PSI 6/8 = 75%). Improved glycated hemoglobin (SMD -0.14 mmol/L, S) and blood glucose (SMD -0.33 mmol/L, S) in B.
Currell 1999 N = 8	8	Nursing record systems	No change in: patient care, patient outcomes. Some administrative benefits.
Kaushal 2003 N = 12	8	Physician order entry and clinical decision support systems	Physician order entry: decrease in serious medication error (PSI 2/5 = 40%), improved in collollary orders (PSI 1/5 = 20%), improved prescribing behaviors (PSI 100%), improved nephrotoxic drug dose and frequency (PSI 1/5 = 20%). Decision support: improved antibiotic-associated medication errors and adverse drug events (PSI 3/7 = 43%), improvement in theophylinne-associated medication errors (PSI 1/7 = 14%).
Mitchell 2001 N = 61	7	Computer systems in primary care	Increased consultation length (SMD range 48–130 seconds). Improved immunization rates (ARR range 8–34%). Reduced test ordering (ARR range 6–75%). Improved patient outcomes (PSI 17/89 = 19%).
Walton 1999 N = 15	8	Computerized decision support on medication prescribing	Blood concentration of drug (AES 0.69, S), time to reach therapeutic concentration (AES – 0.44, S), patient outcomes (PSI5/6 = 83%), cost savings (PSI 2/2 = 100%)

A broad range of computerized services was examined in six reviews, of which all but one were of good methodological quality. None of the reviews had a specific disease focus.

A large review on various computerized information services identified 100 randomized trials, mainly in outpatient care settings [9]. Some interventions focused on providers, such as reminders and computer-assisted treatment planning, while others focused on patients, such as computer-assisted interactive education and patient reminders. Most types of interventions showed positive effects, mainly related to specific a process of care, such as diagnostic test use, preventive services, and number of drug prescriptions. Ten of the fourteen studies that reported on patient outcomes found positive effects.

A later review by some of the same authors identified 40 randomized trials of computerized knowledge management in diabetes care [10]. It showed that computerized prompting (9 studies) led to improved overall guideline adherence. Meta-analysis of studies using home glucose records in insulin dose adjustment (16 studies) documented a decrease in glycated hemoglobin and a decrease in blood glucose. Several computerized patient-education programs improved diet and indicators regarding metabolic control.

Computerized physician order entry and clinical decision support systems were found to have effects on medication error rates and prescribing behaviors [21]. A review by Walton et al [12] focused particularly on computerized support for determining drug dose. It identified 23 comparative studies of which 16 were randomized trials. Seven of 11 studies on drug doses used found reductions, but the overall reduction was not significant in a meta-analysis. Six studies measured unwanted effects of drugs and four found significant reductions. Five of six studies on patient outcomes showed benefits. Only two studies considered costs and one study found cost savings, which resulted largely from reduced hospital stay.

A review on computerized record systems in primary care identified 61 studies, of which 39 randomized trials focused on professional performance and 11 randomized trials on patient outcomes [27]. Immunization rates improved in nine studies that focused mainly on reminder systems. Performance of preventive tasks improved. Four studies found improvements in diabetes management. A number of studies showed that computer support improved prescribing and reduced test ordering with implied cost savings. Use of computers increased the number of patients with reduced diastolic blood pressure in three studies, but did not consistently improve outcomes of anticoagulation therapy in two other studies. Five studies showed that consultation length increased.

A review on nursing care record systems identified eight trials, which suggested that documentation was improved but that process or outcomes of care were not influenced [14]. The reviewers concluded that no evidence was found regarding effects on performance attributable to changes in the record systems.

Overall, it seems that professional performance and patient outcomes can be improved by the implementation of computers in clinical practice settings.

### Quality management (Table 6)

**Table 6:** Quality management

Author, number of studies	Quality score	Focus	Main results
Shortell 1998 N = 3	3	Inpatient and outpatient settings	No change in: all outcomes.
Wagner 2001 N = 4	5	Nursing homes	Qualitative conclusions.

Two reviews on quality management were found; both were of moderate methodological quality. A large review reported on 55 studies on the impact of continuous quality improvement, but only three were randomized trials [34]. Notably, these found no positive effects. A second review focused on nursing homes [41]. It identified four controlled trials of heterogeneous interventions (two of these appeared to comprise professional education). It concluded qualitatively that specific components of quality management were particularly effective, such as specific training, assessment procedures, quality assessment cycles and the assistance of a quality consultant. Overall, the effects of quality management on professional performance and patient outcomes remain uncertain.

### Mixed interventions (Table 7)

**Table 7:** Mixed interventions

Author, number of studies	Quality score	Focus	Main results
Dijkstra 2004 N = 15	7	Organizational change to implement guidelines in hospitals	Improved professional performance (AOR 8.41, NS)
Gilbody 2003 N = unknown	7	Organizational interventions to improve depression management in primary care	Qualitative conclusions.
Hulscher 1999 N = 4	6	Organizational interventions to improve preventive care in general practice	Improved professional performance (ARR range 3–30%, PSI 4/4 = 100%).
Mandelblatt 1995 N = 3	7	Administrative office systems to enhance breast cancer screening	Increase screening rates (ARR range: 19–21%).
Parkes 2000 N = 8	7	Discharge planning from hospital	Reduction in hospital length of stay (WMD 1.01), increased patient satisfaction (PSI 2/2 = 100%. No change in: health outcomes, overall health costs
Renders 2000 N = 9	8	Organizational interventions to improve diabetes care	Qualitative conclusions.
Solomon 1998 N = 26	8	Enabling interventions (administrative structures) to influence use of diagnostic tests by physicians	As single interventions: improved outcomes (PSI 3/5 = 60%). As part of multifaceted interventions: all improved outcomes (PSI range: 75–100%).

Seven reviews, all but one of which were of good methodological quality, combined various organizational interventions (such as described in Table 1) into one group for the analysis and interpretation.

A comprehensive review on implementation of preventive services in primary care found four controlled trials on organizational interventions, such as involvement of nurses and a different way of booking appointments [20]. All showed intended positive effects. A review on improving breast cancer screening identified three randomized trials on change in office administrative systems, which all showed increased use of mammography screening [24]. Discharge planning prior to leaving hospital resulted in a small reduction in hospital length of stay for elderly medical patients, mixed effects on re-admission and no effects on patient outcomes [30]. A review on interventions to improve physicians' use of diagnostic tests found that 'enabling interventions' (administrative structure of test ordering) led to change in a majority of the studies if used alone and in most studies when used in combination with predisposing or reinforcing interventions [36].

A review on interventions to implement guidelines in hospitals found 15 trials, which included an organizational component (other than revision of professional roles) [15]. A meta-regression analysis showed that this component did not contribute to effects on process measures.

A review on interventions to improve the management of diabetes mellitus in primary care and outpatient settings identified nine trials [33]. These interventions focused on change in the medical record system, arrangements for follow-up, involvement of a pharmacist, and multidisciplinary collaboration. The authors conclude that regular prompted recall and review of patients improve diabetes management. Higher treatment adherence and patient recovery, and lower costs, were achieved in patients with depression by "collaborative care", a comprehensive package of interventions that included educational and organizational strategies [17].

## Discussion

This paper examined the evidence of the effectiveness of a broad range of organizational changes in patient care in terms of effects on professional performance, patient outcomes, and costs. We found evidence that professional performance can be improved by enhancement of the professional roles of non-physicians (nurses, pharmacists, etc.) and by computer systems both for reminding and decision support. Patient outcomes were improved by multidisciplinary teams for patient care, integrated care services, and computer decision support. Few studies considered costs, but cost savings were reported from reviews of integrated care services and not consistently for any other organizational changes. There was little evidence of the effectiveness of quality management.

We have not searched exhaustively so it is possible that we have missed relevant reviews. The conclusions need to be regarded as tentative. The lack of a widely accepted taxonomy for organizational interventions is a problem for the examination of their effectiveness. A previous review on organizational change concluded that the available evidence was difficult to locate, even for expert researchers, and may therefore be largely inaccessible to health care managers [7]. There was a range of organizational approaches to improvement that were not explicitly covered by this paper, such as leadership, process redesign, breakthrough series, organizational culture interventions, and organizational learning [2]. We found no systematic reviews focused on these strategies. The use of a 'percentage studies with improvements' (PSI) implies a vote counting method, which has substantial risk for bias and should therefore be interpreted carefully.

This paper shows that a considerable number of rigorous evaluations of organizational changes have been performed, including many controlled trials. Few reviews report on the efficiency of organizational interventions, although many interventions may be primarily targeted at efficiency gains. While further studies are needed, there is some research evidence available to guide decisions. Integrated care services are particularly promising. Their effectiveness may be based on the fact that these are multifaceted interventions that comprise various organizational changes such as revised professional roles, multidisciplinary teams, use of computers systems, and components of quality management. Continued education of health professionals and patient education are usually components of these integrated care services as well. In this way, they can address a wide range of potential barriers for change, which is likely to increase their effectiveness. Further work should focus on analysing the contributions of the specific components in integrated care services, to identify which particularly contribute to their effectiveness.

To allow interpretation by decision-makers in various contexts which strategies to select it is important to provide sufficient background information on the local context in published studies and reviews of these studies. For instance, it may be important whether an improvement is implemented in a small practice (with informal relationships) or in a large hospital department with formalized structures. In future reviews it would be helpful to provide this background information. It may be helpful to have a set of key factors for such descriptions, which are likely to influence change, such as physicians' attitudes regarding a proposed change, organizational structures and financial incentives.
